# Developmental enamel defects and dental anomalies of number and size in children with growth hormone deficiency

**DOI:** 10.1038/s41598-023-41892-x

**Published:** 2023-09-07

**Authors:** Natalia Torlińska-Walkowiak, Katarzyna A. Majewska, Anna Sowińska, Andrzej Kędzia, Justyna Opydo-Szymaczek

**Affiliations:** 1 Department of Pediatric Dentistry, Poznan University of Medical Sciences, 70 Bukowska Street, 60-812 Poznan, Poland; 2 Department of Pediatric Diabetes, Auxology and Obesity, Poznan University of Medical Sciences, 27/33 Szpitalna Street, 60-572 Poznan, Poland; 3 Department of Computer Science and Statistics, Poznan University of Medical Sciences, 7 Rokietnicka Street, 60-806 Poznan, Poland

**Keywords:** Diseases, Endocrinology, Medical research

## Abstract

Growth hormone is meaningfully involved in the processes of tooth cells differentiation and tissue formation. The aim of the study was to evaluate the occurrence of dental anomalies: microdontia, macrodontia, hypodontia and developmental defects of enamel (DDE) amongst a group of isolated growth hormone deficient (GHD) patients and healthy children. This cross-sectional study was based on a group of 101 Caucasian children: 33 with GHD (mean age 10.94, SD 2.51) and 68 being healthy, normal height subjects (mean age 10.4, SD 2.38). The dental examination in primary and permanent teeth was carried out by one trained and calibrated dentist, in accordance with the WHO guidelines. It was observed that 33% of GHD patients suffer from dental anomalies (hypodontia, microdontia or macrodontia), the difference between the study group and the control group was statistically significant (33% vs 4%, *p* < 0.001). Hypodontia and microdontia/macrodontia were the most common problems affecting 18% and 21% of the GHD individuals, respectively. The prevalence of DDE did not differ significantly between GHD group and the control group (58% vs 48%, *p* > 0.05). As children with GHD present more dental anomalies than their healthy coevals, clinicians should be aware of the possible oral health problems associated with GHD and consider dental screening and management as part of the patient’s overall health care plan.

## Introduction

Dentition development is a long-lasting process influenced by multiple genetic and environmental factors. It starts in pregnancy and, apart from wisdom teeth, is continued up to the age of 13 years. During tooth development, permanent teeth, which replace the primary dentition, develop from successional lamina. The mesenchyme surrounding the successional bud is under the control of different molecules, in particular bone morphogenetic proteins 2 and 4 (BMP2 and BMP4)^1^. Transforming growth factors, including BMP4, are among the most important factors in tooth morphogenesis after the family of fibroblast growth factors (FGF)^2^. A study reports associations between a variation in permanent tooth size and genetic polymorphisms in *BMP4, BMP2* indicating a possible role of these genes in dental morphology^3^.

Growth hormone (GH), a crucial regulator of the growth in children, and the growth hormone-insulin-like growth factor I (IGF-I) induce the production of BMP2 and BMP4, and the transforming growth factor-beta superfamily^4^. GH induces preameloblast differentiation and enamel formation, odontoblast differentiation, osteodentin and tubular dentine formation^4, 5^. The effect of GH status on hard tooth tissues was analyzed in an animal model by Smidt et al.^6^. Dwarf animals showed smaller crown, shorter and smaller dentin roots and mesiodistal width at the cementoenamel junction (CEJ). This study implied that GH influences tooth crown and root development prior to dentinogenesis, as well as during the appositional growth of dentin.

Growth failure as a result of growth hormone deficiency (GHD) in children can usually be diagnosed around the age of 2–4 years^7, 8^, while mineralization of permanent crowns takes place up to the age of 8 years. During enamel maturation, a dynamic process with cellular, biochemical, genetic, and epigenetic changes takes place in the developing tissue^9^. The developing dental enamel is highly susceptible to different systemic and local factors and its regeneration after damage appear to be impossible^10^. Signs of development disturbances comprise enamel hypoplasia and hypomineralization^10^.

Data concerning the association between growth hormone deficiency and developmental dental anomalies in children are sparse^11, 12^. Hence, the aim of the study was to evaluate the occurrence of dental anomalies: hypodontia, microdontia/macrodontia, developmental defects of enamel amongst the population of growth hormone deficient patients.

## Methods

A representative sample size was calculated based on the incidence of GHD and demographic data. An estimated samples size was 20 with a 5% margin of error and 95% confidence level, assuming the incidence of GHD 1 per 20,000^13–15^ and the number of children aged 7–17 in Greater Poland Province equal to 400,000. Since GHD is rare, reports on the oral health of patients suffering from this condition seem particularly valuable. Thus, all patients with GHD who matched inclusion criteria were invited to participate in this cross-sectional study.

Thus, the sample finally comprised 33 Caucasian children with isolated idiopathic growth hormone deficiency, patients of the Department of Pediatric Diabetes, Auxology and Obesity of the Poznan University of Medical Sciences. As a control group, we invited 68 healthy Caucasian children who visited the university dental clinic in Poznań (Greater Poland Province) for the control check-up.

Data collection was performed from October 2015 to June 2018. Patients from the study group were of an age ranging from 7.08 to 17.42 years; the mean (SD) age was 10.94 (2.51) years old, and 20 (61.0%) of them were boys and 13 (39.0%) were girls. The mean (SD) age of patients in the control group was 10.47 (2.38) years (Table 1).

**Table 1:** Demographic characteristics of the study and control group.

Variables		Study group (N = 33)	Control group (N = 68)	*P*-value
Gender	F/M	13(39%)/ 20 (61%)	29 (42%)/ 39 (57%)	0.76^a^
Age	Years, mean (SD)	10.94 (2.51)	10.47 (2.38)	0.37^b^
HSDS	Years [median, min–max]	− 2.27 (− 3.35–0.08)	0.27 (− 1.47–1.71)	< 0.001^c^
FT4 (ng/dl)	mean (SD)	1.10 (0.16)	–	–
TSH (µIU/ml)	mean (SD)	2.02 (0.87)	–	–
Age of starting GH therapy (years)	Years, mean (SD)	9.33 (2.62)	–	–
Dentition	Mixed/permanent	24(73%)/9(27%)	47(69%)/ 21(31%)	0.89^d^

^a^Chi-square test.

^b^Student’s t-test.

^c^Mann Whitney test.

^d^Chi-square test with Yates correction.

The subjects from the control group were randomly selected; every third patient, of the appropriate age, fulfilling the inclusion and exclusion criteria and recruited during routine check-up visits in a dental clinic, was invited to join the study. The structure of the control group in terms of age and sex was similar to the study group.

The inclusion criteria for the study group comprised of being over 7 years of age (tooth crown morphogenesis completed); a diagnosis of isolated idiopathic GHD based on a peak of GH levels below 10 ng/ml in two stimulation tests (with insulin, glucagon, or clonidine); a short stature defined as at least 2 SD (standard deviations) below the normal mean height appropriate for the age and gender for GHD children before GH therapy, or a previous short stature and GHD diagnosis in children during GH therapy.

For healthy children from the control group, the inclusion criteria comprised of being of normal height (between the 10th and 90th percentile) and aged over 7 years. Possible factors related to dental abnormalities as traumas, localized infections, irradiation, chemotherapy, perinatal and postnatal problems and malnutrition were excluded.

The exclusion criteria for GHD and healthy patients were as follows: an organic or combined form of GHD, any other diseases apart from GHD, such as genetic syndromes, systemic chronic diseases, other endocrinopathies, malabsorption syndromes, malnutrition or other pathologies related to growth retardation, as well as any oncological diagnosis and treatment in the patient’s history. Individuals from both groups lived in the same region (the Greater Poland Province) under similar environmental conditions.

### Examination

During the medical examination, the height standard deviation score (HSDS- the standard deviation score of a child’s height in relation to gender and age) was calculated in accordance with the guidelines for all the children^16^. Growth hormone secretion in GHD patients was estimated based on a physiological sleep test and two standard stimulation tests during routine diagnostic procedures. Patients with peak levels of GH below 10 ng/ml (DIAsource hGH, IRMA) were diagnosed with GHD.

The dental examination was carried by a qualified pediatric dentist (N.T.W.), who was calibrated before the study by an experienced pediatric dentist (J.O.S.). Calibration exercises were performed on healthy children, apart from the main study. The children were evaluated by N.T.W, who was considered calibrated when her assessments reached a substantial correlation with the evaluations of J.O.S., and considerable correlation of repeated evaluations (Cohen’s Kappa value = 1 for dental anomalies of number and size and > 0.80 for Developmental Defects of Enamel index (DDE)).

The children sat on a straight backed chair facing the examiner, illumination was provided by a portable lamp in accordance with the World Health Organization guidelines^17^. Prior to the oral examination, the surfaces of the teeth were wiped with a gauze to remove any debris that was present. All erupted teeth, both primary and permanent, were examined. Findings were recorded manually onto a specially designed data recording form.

Microdontia was identified by direct observation and based on visual judgment when the mesiodistal crown diameter was smaller than the same dimension of the opposing or contralateral tooth in the same patient^18^. Conical phenotype or peg-shaped maxillary lateral incisors were also included^19, 20^. Macrodontia was diagnosed when when the mesiodistal width of the crown was bigger than the same dimension of the opposing or contralateral tooth^12^.

Hypodontia was suspected when the clinical oral examination confirmed the lack of an erupted tooth in the expected time frame, the persistence of a primary tooth in the arch beyond the anticipated date of the eruption of its successor, or the asymmetric loss of primary teeth and an interview with parents verifying such information to exclude extractions due to orthodontic or any other reasons. In all the cases of hypodontia, parents reported earlier diagnosed dental agenesis and had to present their juvenile x-rays to confirm tooth agenesis. Data on the timing, the sequence of teeth exfoliation and emergence for the Polish population were considered^21^.

The diagnostic criteria for enamel defects were based on the modified version of the Developmental Defects of Enamel index (DDE) for use in screening surveys^22^. Three basic types of enamel detects were identified: demarcated opacities, diffuse opacities and hypoplasia.

### Statistical analysis

Statistical analysis was performed using Dell Inc. (2016) Dell Statistica (a data analysis software system), version 13^23^.

The distribution of data was evaluated by the Shapiro Wilk test. Quantitative data were expressed as the mean (SD) for normally distributed variables or median (range) for non-normally distributed variables. The Student’s t-test and Mann–Whitney U test were used to compare the differences between unpaired quantitative data normally and not-normally distributed, respectively. Categorical variables were presented as percentages and compared by the chi-square test, the chi-square test with Yates correction, Fisher’s exact test, and the Fisher–Freeman–Halton test.

A *P* value < 0.05 was considered significant.

### Ethical approval

All procedures performed in the study were in accordance with the 1964 Helsinki declaration and its later amendments. The research protocol was approved by the Bioethics Committee at the Poznan University of Medical Sciences (Resolution No. 785/15).

### Informed consent

Guardians of all participants under the age of 16 provided informed consent to the research. Informed Consent was also obtained from the adolescents above 16 years of age.

## Results

Table 1 presents the demographic characteristics of the study group and the control group. There were no differences in terms of age and sex between the study and control groups. Children from the study group had HSDS statistically significantly lower than children from the control group (*p* < 0.001). All healthy children had HSDS within the normal range. Children in the study group commenced GH treatment at the mean (SD) age of 9.33 (2.62) years.

Table 2 shows the distribution of tooth anomalies. There was a statistically significant difference in the percentage of children with abnormal tooth morphology between the study group and the control group (*P* < 0.001). It was observed that 33% of the GHD group suffer from hypodontia or alteration in tooth size (microdontia or macrodontia). Most often 2 teeth (45%) were affected by anomalies in the GHD group, less often 1 tooth (36%). Two children from the GHD group had 4 teeth affected.

**Table 2:** The distribution of dental anomalies concerning the number and size of teeth in the study and control groups.

	Study group N = 33 (100%)	Control group N = 68 (100%)
Number (%) of subjects with dental anomaly of number and/or size	11 (33%)	3 (4%)^a^
Number (%) of affected teeth per child	N = 11 (100%)	N = 3 (100%)
1 tooth	4 (36%)	0 (0%)
2 teeth	5 (45%)	3 (100%)
4 teeth	2 (18%)	0 (0%)
Number (%) of subjects with hypodontia	6 (18%)	1 (2%)^b^
Number (%) of subjects with hypodontia affecting	N = 6(100%)	N = 1(100%)
Permanent lateral incisor	2 (33%)	0 (0%)
Permanent canine	1 (17%)	0 (0%)
Primary canine	1 (17%)	0 (0%)
Second premolar	3 (33%)	1 (100%)
Number (%) of subjects with tooth size anomaly	7 (21%)	2 (3%)^c^
Number (%) of subjects with tooth size anomaly affecting	N = 7 (100%)	N = 2 (100%)
Microdontic central incisor	2 (29%)	0(0%)
Microdontic lateral incisor	4 (57%)	0 (0%)
Macrodontic central incisor	1 (14%)	0 (0%)
Macrodontic second premolar	0 (0%)	1 (50%)
Microdontic second molar	0 (0%)	1 (50%)
Number (%) of affected teeth by size anomaly per child	N = 7(100%)	N = 2(100%)
1 tooth	2 (29%)	0 (0%)
2 teeth	5 (71%)	2 (100%)
Number (%) of subjects with microdontia	6 (18%)	1 (2%)^d^

^a^*P* < 0.001 as compared to the percentage of subjects with dental anomalies in the study group.

^b^*P* = 0.007 as compared to the percentage of subjects with hypodontia in the study group.

^c^*P* = 0.008 as compared to the percentage of subjects with size anomaly in the study group.

^d^*P* = 0.005 as compared to the percentage of subjects with microdontia in the study group.

There was a statistically significant difference in the percentage of subjects with missing teeth between the groups. In the GHD group, 6 patients (18%) reported to have a missing tooth, compared to 1 person (2%) in the control group. Second premolars were missing most often (in 3 individuals), followed by permanent lateral (in 2 individuals), and one primary and one permanent canine.

A statistically significant difference was found between the percentage of GHD children and healthy peers affected by an alteration of tooth size (21% vs 3%, *P* = 0.008). In the study group the anomaly affected one tooth in 2 children and two teeth in 5 children.

Single tooth microdontia without an additional number and size anomalies was noted in 6 subjects (18%) in the GHD group.

There was not any statistically significant difference between the groups regarding the occurrence of enamel defects (Table 3).

**Table 3:** Prevalence of developmental enamel defects in GHD children and healthy age-mates.

	Study group N = 33 (100%)	Control group N = 68 (100%)	*P*-value
Number (%) of subjects with any type of defect	19 (58%)	33 (48%)	0.39
Number (%) of subjects with demarcated opacity	19 (58%)	33 (48%)	0.39
Number (%) of subjects with diffuse opacity	9 (27%)	24 (35%)	0.42
Number (%) of subjects with hypoplasia	0 (0%)	5 (7%)	0.32

## Discussion

To what extent GH influences tooth development is difficult to estimate because both environmental and genetic factors can lead to dental anomalies. Up to now, more than 200 genes involved in embryonic development, morphogenesis, and differentiation of teeth processes have been identified^2^.

Amongst the causative agents of enamel defects are environmental intoxications such as fluoride and dioxins, trauma, localized infections, irradiation, chemotherapy, infectious diseases, perinatal and postnatal problems, and malnutrition^24^.

According to a recently published systematic review, data concerning the oral health of patients with GHD are limited^25^. Most studies describe the influence of GH deficiency and GH therapy on the proper bone mineralization and development of craniofacial structures^7, 26–28^. Available information on dental anomalies in GHD is based mainly on case reports, three of which describe patients with coexisting solitary median incisor, hypopituitarism and other midline defects such as a cleft lip and palate^29–31^, two cases of the melange of GHD and amelogenesis imperfecta^32, 33^, and one case of an adult woman with GHD, microdontia, and impacted permanent teeth^34^.

Congenital dental agenesis is a common dental anomaly that typically affects otherwise healthy individuals, but it has also been associated with more than 150 syndromic diseases^2^. The non-syndromic dental agenesis usually affects only one or two teeth^35, 36^ and the most commonly missing teeth are premolars and permanent maxillary lateral incisors^36, 37^ what was also confirmed in our study.

The overall frequency of patients with congenitally missing permanent teeth is reported in recent investigations as up to 10% in various studies from different countries^1, 35, 38^. In the study by Kielan-Grabowska et al. 11.6% of Polish orthodontic patients showed hypodontia of permanent dentition^39^. There is little data available in literature on the prevalence of hypodontia in children with growth hormone deficiency. Sarnat et al.^40^ examined a group of 19 patients with isolated GHD and a group of 13 patients with Laron-type dwarfism, and found hypodontia in 30% of the patients. In our GHD group 6 children (18%) showed hypodontia, which affected second premolars, permanent lateral incisors, permanent and primary canine. In the control group only one child had one premolar congenitally missing.

Unfortunately, our observations could not be confirmed in all subjects by dental radiographs, which is an important limitation. In all cases of hypodontia, the parents confirmed an earlier diagnosis of congenital tooth absence. We additionally tried to rule out possible diagnosis caused by teeth impaction, early extraction or trauma, by conducting a detailed interview with the parents. On the other hand, the observed prevalence of hypodontia in patients during early mixed dentition stage might be underestimated. Without obvious clinical signs, parents of children who were not referred for dental x-rays before, could have been unaware of the possible absence of later erupting teeth.

The congenital absence of primary teeth is less frequent compared to secondary dentition with a prevalence between 0.1 and 1.5%^1, 41^. Primary teeth agenesis often, but not always, correlates with the absence of their permanent successors^2^. Only one patient from our study group was missing an anterior primary tooth. This seems to be in line with other research studies, proving that it is a rare abnormality^42^. Unusually, it was a canine, while according to literature, the maxillary lateral incisors account for over 50% and, together with mandibular incisors for 90% of all affected primary teeth^2^.

In many studies, females were found to have a higher prevalence of dental agenesis than males^2, 43, 44^. According to Rakhshan et al. the higher rates observed in females might be associated with smaller jaws and lack of space for the development of dental germs^45^. This phenomenon could be also one of the possible explanations of the high prevalence of dental agenesis observed in GHD children as skeletal and dental age discrepancy in GHD children may be accompanied by a problem of space for developing teeth^28^. In fact, our research involved more male than female patients, because male predominance is typical for GHD studies^46^. As a consequence, also more boys than girls presented agenesis.

Alterations of tooth size are less common than dental agenesis and they may also affect both primary and permanent teeth^35^. Information regarding the prevalence of micro- and macrodontia in healthy children is scarce and it is difficult to compare the results of different studies due to the varying criteria used in assessments and different populations studied^47^. The most commonly involved tooth is the maxillary lateral incisor^37^, which may also be shaped like an inverted cone (a “peg-shaped lateral”). Peg-shaped lateral incisors are considered as a different phenotypical expression of the same genotype as dental agenesis. In our study, 7 (21%) GHD children had tooth size anomaly compared to only 2 (3%) healthy subjects, while 6 (18%) had significantly reduced teeth dimensions compared to only 1 individual from the control group. Although microdontia is considered a characteristic feature of growth hormone deficiency^4^, literature data on the prevalence of this kind of abnormality in GHD children are limited. Oliveira-Neto et al.^48^ observed a reduction in dental mesiodistal width in untreated adults with GH deficiency. In patients with pituitary dwarfism examined by Bigeard et al.^11^ premolar crowns had significantly reduced dimensions, while the other teeth were of normal size. Our subjects with GHD showed tooth size anomalies of permanent incisors.

One could argue that there are interpopulation and regional variations in the crown size, with larger crown widths observed in Africans, intermediate in Asians, and much smaller in Europeans^49^. However, our patients were a homogenous group with the same ethnic, cultural, demographic, and regional origin, which reduces bias due to this diversity.

Congenitally missing teeth and microdontia lead to problems with space, malocclusion, esthetic issues and psychological problems. Dental agenesis may result in the misplacement of teeth, periodontal damage or inadequate bone height in the upper and lower jaw^50^.

There are no data in literature on the frequency of developmental enamel defects in children with growth hormone deficiency^25^.The potential association of dental developmental defects and hormones receptors was investigated by Arid et al.^51^. It was demonstrated that genetic polymorphism in GH receptors (rs1509460) is associated with alterations in the ameloblast function and developmental defects of enamel. In a rat incisor model both inner and outer enamel epithelium were positive for GH receptors in the bell stage of tooth development. Ameloblast apoptosis was associated with down-regulated expression of the insulin-like growth factor-1 receptor. These findings support the premise that GH and IGF-I may play a role in embryonic tooth development by regulating the epithelial-mesenchymal interactions that influence events in growth and cytodifferentiation^52^.

Our present study revealed that 58% of GHD were affected by DDE compared to 48% of the control group and the difference was not statistically significant. Disturbances in amelogenesis are associated with poor esthetics and a higher susceptibility of teeth to harmful local factors^10^, but growth hormone deficient individuals do not seem more vulnerable than healthy children.

## Conclusions

The study confirms a significant association between GHD and dental anomalies of both number and size. Children with GHD had a significantly higher prevalence of microdontia and hypodontia than their healthy coevals, which may have a negative impact on oral health and esthetics. At the same time, the prevalence of enamel defects in the study and control groups did not differ significantly. Further research is needed to understand the underlying mechanisms and to develop management strategies for dental anomalies in patients with GHD. Clinicians should be aware of the possible oral health problems associated with GHD and consider dental screening and management as part of the patient’s overall health care plan.

## Data Availability

The data that support the findings of this study are available on reasonable request from the corresponding author.

## References

[1] Juuri E, Balic A (2017). The biology underlying abnormalities of tooth number in humans. J. Dent. Res..

[2] Abu-Hussein M, Watted N, Yehia M, Proff P, Iraqi F (2015). Clinical genetic basis of tooth agenesis. J. Dent. Med. Sci..

[3] Gerber JT, Dos Santos KM, Brum BK (2021). Odontogenesis-related candidate genes involved in variations of permanent teeth size. Clin. Oral Investig..

[4] Litsas G (2015). Growth hormone and craniofacial tissues. An update. Open Dent. J..

[5] Li H, Bartold PM, Zhang CZ (1998). Growth hormone and insulin-like growth factor I induce bone morphogenetic proteins 2 and 4: A mediator role in bone and tooth formation?. Endocrinology.

[6] Smid JR, Rowland JE, Young WG (2007). Mouse molar dentin size/shape is dependent on growth hormone status. J. Dent. Res..

[7] Funatsu M, Sato K, Mitani H (2006). Effects of growth hormone on craniofacial growth. Angle Orthod..

[8] Cantu G, Buschang PH, Gonzalez JL (1997). Diferential growth and maturation in idiopathic growth-hormone-defcient children. Eur. J. Orthod..

[9] Jeremias F, Koruyucu M, Küchler EC (2013). Genes expressed in dental enamel development are associated with molar-incisor hypomineralization. Arch. Oral Biol..

[10] Opydo-Szymaczek J, Gerreth K, Borysewicz-Lewicka M (2018). Enamel defects and dental caries among children attending primary schools in Poznań, Poland. Adv. Clin. Exp. Med..

[11] Bigeard L, Sommermater J (1991). Retard dentaire et microdontie chez l'enfant atteint de déficit en hormone somatotrope [dental delay and microdontia in children with somatotropin hormone deficiency]. J. Biol. Buccale.

[12] Stolbizer F, Cripovich V, Paolini A (2020). Macrodontia associated with growth-hormone therapy: A case report and review of the literature. Eur. J. Paediatr. Dent..

[13] Sharma H, Mathur SK, Purwar N (2021). Clinical and biochemical phenotype of Indian children with different types of idiopathic growth hormone deficiency and their association with pituitary height on MRI. Indian J. Endocr. Metab..

[14] Stochholm K, Gravholt CH, Laursen T (2006). Incidence of GH deficiency: A nationwide study. Eur. J. Endocrinol..

[15] Thomas M, Massa G, Craen M (2004). Prevalence and demographic features of childhood growth hormone deficiency in Belgium during the period 1986–2001. Eur. J. Endocrinol..

[16] Kułaga Z, Litwin M, Tkaczyk M (2011). Polish 2010 growth references for school-aged children and adolescents. Eur. J. Pediatr..

[17] World Health Organization (1997). Oral Health Surveys: Basic Methods.

[18] Chen Y, Zhou F, Peng Y, Chen L, Wang Y (2019). Non-syndromic occurrence of true generalized microdontia with hypodontia: A case report. Medicine (Baltimore).

[19] Choi SJ, Lee JW, Song JH (2017). Dental anomaly patterns associated with tooth agenesis. Acta Odontol. Scand..

[20] Shalish M, Peck S, Wasserstein A, Peck L (2010). Increased occurrence of dental anomalies associated with infraocclusion of deciduous molars. Angle Orthod..

[21] Szydłowska-Walendowska B, Wochna-Sobańska M (2005). Dates and sequence of the eruption of permanent teeth in children from Łódź. Czas Stomat.

[22] Clarkson J, O’Mullane MD (1989). A modified DDE index for use in epidemiological studies of enamel defects. J. Dent. Res..

[23] TIBCO Software Inc. Statistica (data analysis software system), version 13 (2017).

[24] Wong HM (2014). Aetiological factors for developmental defects of enamel. Austin J. Anat..

[25] Torlińska-Walkowiak N, Majewska KA, Kędzia A, Opydo-Szymaczek J (2021). Clinical implications of growth hormone defciency for oral health in children: A systematic review. J. Clin. Med..

[26] Alatzoglou KS, Webb EA, Le Tissier P, Dattani MT (2014). Isolated growth hormone deficiency (GHD) in childhood and adolescence: Recent advances. Endocr. Rev..

[27] Choi SH, Fan D, Hwang MS, Lee HK, Hwang CJ (2017). Effect of growth hormone treatment on craniofacial growth in children: Idiopathic short stature versus growth hormone deficiency. J. Formos. Med. Assoc..

[28] Torlińska-Walkowiak N, Majewska KA, Sowińska A, Kędzia A, Opydo-Szymaczek J (2022). Skeletal and dental age discrepancy and occlusal traits in children with growth hormone deficiency and idiopathic short stature. Clin. Oral Investig..

[29] Dutta D, Jain R, Kumar M (2013). Solitary median maxillary central incisor, a clinical predictor of hypoplastic anterior pituitary, ectopic neurohypophysis and growth hormone deficiency. J. Pediatr. Endocrinol. Metab..

[30] Rodríguez Ogando A, Roldán Martín MB, Rodríguez Arnao MD, Rodríguez Sánchez A (2011). Panhipopituitarismo congénito como parte del síndrome de incisivo único medial [Congenital panhypopituitarism as part of the solitary median incisor syndrome]. An. Pediatr. (Barc.).

[31] Bretéché F, Delaire J, Ginguene Y (1984). Agénésie de l'incisive centrale supérieure. Ses rapports avec le nanisme hypophysaire [Agenesis of the superior central incisor. Its relation to pituitary nanism]. Rev. Stomatol. Chir. Maxillofac..

[32] Dündar B, Erçal D, Böber E, Berk T, Büyükgebiz A (2002). Amelogenesis imperfecta with growth hormone deficiency in a 12 year-old boy. J. Pediatr. Endocrinol. Metab..

[33] Sharma S, Antarmayee P (2016). Familial amelogenesis imperfecta with growth hormone deficiency and skin lesions—Case report of unique melange of disorders. J. Appl. Dent. Med. Sci..

[34] Ferrante F, Blasi S, Crippa R, Angiero F (2017). Dental abnormalities in pituitary dwarfism: A case report and review of the literature. Case Rep. Dent..

[35] Souza-Silva BN, de Andrade Vieira W, de Macedo Bernardino I (2018). Non-syndromic tooth agenesis patterns and their association with other dental anomalies: A retrospective study. Arch. Oral Biol..

[36] Song JS, Shin TJ, Kim YJ (2018). Prediction of agenesis of the mandibular second premolar using the developmental stages of the mandibular canine, first premolar, and second molar. Arch. Oral Biol..

[37] Mostowska A, Biedziak B, Zadurska M (2018). GREM2 nucleotide variants and the risk of tooth agenesis. Oral Dis..

[38] Polder BJ, Van’t Hof MA, Van der Linden FP, Kuijpers-Jagtman AM (2004). A meta-analysis of the prevalence of dental agenesis of permanent teeth. Community Dent. Oral Epidemiol..

[39] Kielan-Grabowska Z, Kawala B, Antoszewska-Smith J (2019). Hypodontia—not only an orthodontic problem. Dent. Med. Probl..

[40] Sarnat H, Kaplan I, Pertzelan A, Laron Z (1988). Comparison of dental findings in patients with isolated growth hormone deficiency treated with human growth hormone (hGH) and in untreated patients with Laron-type dwarfism. Oral Surg. Oral Med. Oral Pathol..

[41] Lochib S, Indushekar KR, Saraf BG, Sheoran N, Sardana D (2015). Occlusal characteristics and prevalence of associated dental anomalies in the primary dentition. J. Epidemiol. Glob. Health.

[42] Carvalho JC, Vynckier F, Declerck D (1998). Malocclusion, dental injuries and dental anomalies in the primary dentition of Belgian school children. Int. J. Paediatr. Dent..

[43] Tallón-Walton V, Nieminen P, Arte S (2010). An epidemiological study of dental agenesis in a primary health area in Spain: Estimated prevalence and associated factors. Med. Oral Patol. Oral Cir. Bucal..

[44] Khalaf K, Miskelly J, Voge E, Macfarlane TV (2014). Prevalence of hypodontia and associated factors: A systematic review and meta-analysis. J. Orthod..

[45] Rakhshan V (2015). Congenitally missing teeth (hypodontia): A review of the literature concerning the etiology, prevalence, risk factors, patterns and treatment. Dent. Res. J. (Isfahan).

[46] Majewska KA, Kedzia A, Kontowicz P (2020). Polymorphism of the growth hormone gene GH1 in Polish children and adolescents with short stature. Endocrine.

[47] Hölttä P, Alaluusua S, Saarinen-Pihkala UM, Peltola J, Hovi L (2005). Agenesis and microdontia of permanent teeth as late adverse effects after stem cell transplantation in young children. Cancer.

[48] Oliveira-Neto LA, Nascimento JKF, Salvatori R (2022). Growth of teeth and bones in adult subjects with congenital untreated isolated growth hormone deficiency. Growth Horm. IGF Res..

[49] Hikita Y, Yamaguchi T, Tomita D (2018). Growth hormone receptor gene is related to root length and tooth length in human teeth. Angle Orthod..

[50] Eliacik BK, Atas C, Polat GG (2021). Prevalence and patterns of tooth agenesis among patients aged 12–22 years: A retrospective study. Korean J. Orthod..

[51] Arid J, Oliveira DB, Evangelista SS (2019). Oestrogen receptor alpha, growth hormone receptor, and developmental defect of enamel. Int. J. Paediatr. Dent..

[52] Joseph BK, Savage NW, Young WG, Waters MJ (1994). Prenatal expression of growth hormone receptor/binding protein and insulin-like growth factor-I (IGF-I) in the enamel organ. Role for growth hormone and IGF-I in cellular differentiation during early tooth formation?. Anat. Embryol. (Berl.).

